# Guiding attention in the classroom: An eye‐tracking study on the associations between preservice teachers' goals and noticing of student interactions

**DOI:** 10.1111/bjep.12748

**Published:** 2025-03-10

**Authors:** M. Daumiller, R. Böheim, A. Alijagic, D. Lewalter, A. Gegenfurtner, T. Seidel, M. Dresel

**Affiliations:** ^1^ Ludwig‐Maximilians‐Universität München Munich Germany; ^2^ Technical University of Munich Munich Germany; ^3^ University of Augsburg Germany

**Keywords:** eye tracking, motivation, noticing, student‐oriented goals, teacher professional vision

## Abstract

**Background:**

Teachers' goals play an important role in teaching quality and student outcomes. However, the processes through which this aspect of teacher motivation translates into specific teaching behaviours remain unclear.

**Aims:**

This study investigates how goals directed at students and the classroom are associated with visual information processing of classroom events, aiming to link teacher motivation with professional vision.

**Sample:**

The study involved 51 preservice teachers with an average of 36 days of practical teaching experience.

**Methods:**

Participants' eye movements were recorded through eye tracking while they observed a video stimulus of an 11th‐grade mathematics classroom. Through an interview, participants specified their goals for individual students and the whole classroom after having watched the start of the video stimulus. During the rest of the 3‐min‐long simulation, eye‐tracking recorded the number and duration of fixations on students.

**Results:**

Goals directed at individual students were associated with more and longer fixations. In contrast, goals targeting the entire classroom were associated with shorter fixation durations on individual students, indicating a more even distribution of visual attention. Especially mastery goals drove these patterns; nuanced effects were observed depending on goal content and the visual saliency of student behaviours.

**Conclusions:**

Preservice teachers' student‐oriented goals shape their visual attention in the classroom, influencing how they perceive the interaction with students. This research highlights the importance of integrating teacher motivation with professional vision to understand the cognitive pathways that link motivation to teaching behaviours. The study also demonstrates the utility of eye tracking technology in exploring these processes.

## INTRODUCTION

It has already been established that teacher motivation matters for teaching quality and student outcomes—however, the processes that explain why certain components of teacher motivation lead to specific teaching behaviours are still largely unclear (Bardach & Klassen, 2021; Zee & Koomen, 2016), calling for specific investigations thereof (Lazarides et al., 2024). Further, research on teacher goals and teacher professional vision (Seidel & Stürmer, 2014) has not yet been linked in systematic ways, although theoretically strong associations could be assumed. With the present research, we seek to close this gap, showing that teachers' goals affect visual attention allocated to classroom events.

In the pathway from teacher motivation to teaching behaviours, Daumiller et al. (2022) recently introduced teachers' goals directed at their students as mediators between components of teachers' personal motivation and their teaching behaviours. Unlike goals focused on oneself (teachers' self‐directed achievement goal orientations; Butler, 2007; Nitsche et al., 2011), these are goals directed at their students (Daumiller et al., same issue; Daniels et al., 2013). Butler (2007) already highlighted the importance of such goals, noting that teachers' goals are largely defined by their students' achievement and well‐being. These goals can focus specifically on individual students (e.g. wanting a particular student to master a task) or the whole classroom (e.g. aiming for the whole class to improve their social ties). From a cognitive psychology perspective, goals are believed to shape the perception and processing of classroom events by selectively directing attention, in particular, visual attention. Such a conscious directing of attention to specific, learning‐relevant interactions is termed *noticing* in professional vision research, where the use of eye tracking is a novel and increasingly important assessment approach (for a meta‐analytic review, see Keskin et al., 2024). While differences in noticing have often been attributed to differences in knowledge, the role of teacher motivation has been largely overlooked (Seidel et al., 2021, 2024; Seidel & Stürmer, 2014) and, surprisingly, the impact of goals on attentional processes has scarcely been analysed in educational contexts (cf. Kücherer et al., 2021).

With the present research, we aim to reduce this gap by investigating teachers' noticing as a processual and cognitive consequence of teachers' pursuit of goals directed at their students and the classroom. By recording the eye movements of preservice teachers while they observe an authentic video of a classroom interaction, we study the effects of motivation on noticing and the importance of teachers' goals for the situational guidance of visual attention in instructional settings (van Es & Sherin, 2021).

### Teachers' goals for individual students and the classroom

Understanding the motivation behind teaching behaviours is crucial for enhancing instructional practices. Teachers' motivation influences not only their own professional experiences but also students' educational outcomes (Fives & Buehl, 2016; Lauermann & Butler, 2021), with teaching behaviours serving as a key pathway linking teacher motivation to student outcomes (Daumiller et al., 2022; Frenzel et al., 2009; Lauermann & Butler, 2021; Lazarides & Schiefele, 2021; Zee & Koomen, 2016). However, the mechanisms by which teacher motivation translates into specific teaching behaviours remain unclear and require specific investigation (Bardach & Klassen, 2021; Lazarides et al., 2024; Zee & Koomen, 2016).

Teachers' achievement goals are central aspects of their motivation (e.g. Butler, 2007; Nitsche et al., 2011) that have been found to explain a wide variety of their teaching behaviours (for recent overviews, see Daumiller et al., 2022; Han & Gao, 2023). Goals are defined as cognitive representations of desired outcomes or end states that individuals strive to achieve through their behaviour (Locke & Latham, 1990). In the context of achievement motivation, goals serve as guiding frameworks that organize and direct attention, effort, and persistence (Elliot & Fryer, 2008). They entail rather stable and habitualized orientations and state‐like goals that are actualized in specific teaching situations and responsible for the direct impacts of goal setting (Daumiller et al., 2023; Praetorius et al., 2014). Regarding the pathways from teacher motivation to teaching behaviour, Daumiller et al. (2022) showed that besides teachers' self‐directed goals (e.g. ‘My goal is to improve my own skills as a teacher’), the goals that teachers hold for their students (e.g. ‘My goal is that my students improve their skills’) have a relevant role in explaining the cognitive processes linking teacher motivation to teaching behaviour. They also provided evidence that student‐oriented goals can mediate the effects of self‐directed goals and self‐efficacy, thus encouraging the investigation of student‐oriented goals as likely more proximal to their instructional behaviours. To investigate the effects of teachers' goals on professional vision in a specific classroom situation, it accordingly makes sense to focus on their student‐oriented goals therein. Such goals can be directed at single students, groups of students, or the whole classroom (e.g. wanting students to master the course material, score well in examinations, to experience well‐being in class). Paralleling research on teachers' self‐directed goals, an achievement goal approach has fruitfully been used to describe the different types of such goals and their effects. More specifically, researchers have typically distinguished between mastery goals (focused on the development of competences and mastering tasks) and performance goals (focused on performance relative to and as perceived by others), while additionally relational goals (focused on interpersonal relationships with relevant others) and work avoidance goals (focused on getting through the day with little effort) are also considered (Butler, 2007, 2012; Daumiller et al., 2022; Papaioannou & Christodoulidis, 2007; Retelsdorf & Günther, 2011; Watt et al., 2021). Goals directed at students have typically been distinguished based on mastery and performance goals, for which first evidence shows that they relate to differences in teaching behaviours (Daumiller et al., this issue; Daniels et al., 2013; Daumiller et al., 2022).

For example, Daumiller et al. (2022) found that teachers' mastery goals positively predicted the pursuit of student‐oriented mastery goals (i.e. aiming for students to continue learning and improving), which in turn positively affected students' reports of teachers' mastery practices. Student‐oriented performance goals in turn were negatively associated with reports of teachers' mastery practices in the classroom. Likewise, Daumiller et al. (this issue) provide evidence for teachers' student‐oriented goals mattering for teachers' subsequent reports of teaching practices in standardized lesson diaries. In these works, student‐oriented goals were distinguished based on mastery versus performance (see also Daniels et al., 2013). Note that besides these two types of achievement goals, further goals might also be of relevance, for example, goals directed at students' discipline or well‐being in the classroom. While not centred around students' competence (which lies at the heart of the achievement goal approach), such goals may also be meaningful drivers for teachers' classroom behaviours.

But why exactly do such goals go along with different instructional practices? To answer this question, Lazarides et al. (2024) emphasize the critical role of investigating mediating factors, including cognitive processes that we focus on here. Specifically, goals should guide and shape attention and visual processes in the classroom, as outlined next.

### Goals and noticing: Combining motivation and professional vision research

Teachers' goals can be assumed to be strongly interrelated with the visual information processing of classroom events (Seidel et al., 2024). It is well‐known in cognitive psychology that, on a fundamental level, (visual) attention is a process that selects and biases the flow of incoming information and internal representations in the service of effective goal achievement (Dijksterhuis & Aarts, 2010). Goals are of pivotal importance for endogenous (top‐down) attentive processes—they determine both the amount and duration of attention devoted to incoming information (depending on their relevance for goal attainment; Corbetta & Shulman, 2002). Thus, the content of attention refers to the goals and schemata that are active at a specific moment in time, and attention translates goals into overt behaviour (Monsell & Driver, 2000). Surprisingly, the effects of teachers' goals on their attentive processes were seldomly analysed (for one of the few exceptions in a professional development context, see Kücherer et al., 2021)—and not yet considered as a cognitive process mediating the effects of teacher motivation on teaching quality.

Interactions in classrooms are complex, with numerous events occurring simultaneously, requiring teachers to decide what to attend to and what to ignore. Research on teacher professional vision has shown that the ability to direct visual attention to critical aspects of teaching and learning, while disregarding less relevant aspects, depends on the specific teaching situation and develops with increasing professional expertise (Blömeke, 2024; Santagata et al., 2021; Seidel et al., 2021; Stahnke et al., 2016; van Es & Sherin, 2021). The ability to selectively focus attention on relevant events is usually referred to as noticing (Santagata et al., 2021; Seidel & Stürmer, 2014; Stahnke et al., 2016). Grounded in cognitive models of professional vision (Gegenfurtner et al., 2023; Seidel et al., 2024), noticing is described as a cognitive‐psychological filtering process that is shaped by top‐down (goals and intensions derived from professional knowledge and experience) and bottom‐up (saliency of visual stimuli) influences (Gegenfurtner et al., 2011). More specifically, based on cognitive models of information processing (e.g. Schneider & Shiffrin, 1977; Baddeley & Hitch, 2019; Mayer, 2014), three basic cognitive processes come into play: selecting relevant information, organizing it into mental models, and aligning and integrating it with prior knowledge. Due to capacity limitations in the cognitive system (Sweller, 2003), attention must be selectively directed towards the most relevant information, filtering out extraneous details. The perception and interpretation of visual information therefore depend on the quantity and structure of prior knowledge, along with cognitive schemas stored in long‐term memory, which together facilitate the processing of incoming information.

Past research suggests that differences in teachers' ability to notice relevant events are related to differences in teacher expertise, especially regarding their professional knowledge (Farrell et al., 2022; Gabel et al., 2023; Grub et al., 2020; Keskin et al., 2024; Kosel et al., 2023; Martin et al., 2023; Seidel et al., 2021, 2024; Stahnke & Gegenfurtner, 2024; Wolff et al., 2016). Reasons to notice and to decide what is relevant depend on the specific teaching situation, and it is therefore assumed that the situation‐specific goals that teachers set and follow are central to explaining the noticing process. Indeed, several scholars emphasize additional aspects beyond teacher knowledge, including beliefs, metacognition, self‐regulation, and motivational factors such as goals to explain teachers' noticing of what is relevant in the classroom (Blömeke et al., 2015; Gegenfurtner et al., 2020; Schoenfeld, 2011; Seidel et al., 2024)—empirically, motivational aspects, however, have so far largely been ignored.

### Studying teachers' noticing through videos of classroom interaction and eye tracking

In recent years, eye tracking has become a widely used method for assessing teacher noticing (for review, see Grub et al., 2020; Keskin et al., 2024) as it allows researchers to collect non‐self‐reported, processed data on teachers' attention allocation. Among the different metrics, eye fixations are commonly used to assess attention. Based on the eye‐mind‐assumption (Just & Carpenter, 1980), the location and duration of fixations serve as a proxy of what information is being selected and how deep that information is being processed (Van Gog & Jarodzka, 2013). During fixations, the eye is almost motionless, and information is retrieved from the respective visual stimulus (Holmqvist et al., 2011). In their systematic reviews, Grub et al. (2020) and Keskin et al. (2024) reported that the number and duration of fixations were the two most used eye tracking metrics to assess teacher noticing.

Eye tracking studies have further provided informative insights on how teachers attend to individual students. Teachers differ in their capability to monitor several students at the same time or allocate their attention towards struggling students who require more pedagogical support than others (Chaudhuri et al., 2022; Kosel et al., 2021; Seidel et al., 2021). Among these processes, salient on‐task student behaviours, such as asking questions, raising hands, or participating in classroom discussions, significantly attract preservice teachers' attention. For example, Wolff et al. (2016) found that preservice teachers tend to focus their attention on students showing disruptive behaviour, while more experienced teachers are able to maintain an overview of the entire classroom interaction. In contrast, passive students are frequently overlooked by preservice teachers (Gabel et al., 2024; Goldberg et al., 2021). Based on expert‐novice paradigms, these studies show that teachers' knowledge‐based noticing develops with increasing expertise (Blömeke, 2024; Gegenfurtner, 2024; Seidel et al., 2024; Stahnke et al., 2016). From this perspective, the top‐down regulated distribution of attention towards students is considered more characteristic of expert teachers. However, research indicates a considerable variability within expert groups and shows that preservice teachers may also demonstrate expert‐like gaze patterns (i.e. more fixations and shorter fixation durations; e.g. Grub et al., 2022; Schnitzler et al., 2020). This suggests that the top‐down regulated distribution of attention can likely also be influenced by factors beyond professional knowledge such as preservice teachers' underlying intentions, plans, or goals.

### The present research

With the present work, we seek to link teacher goals with teacher professional vision, aiming to show how teachers' goals in a given teaching situation guide the visual processing of student behaviours and classroom events, which ultimately give rise to leveraged professional teaching practices. As outlined before, teachers are driven by a plethora of goals that can be classified on various levels. In this work, we focus on teachers' student‐oriented goals that should be more proximal to teaching behaviours than underlying self‐directed achievement goals. As such, if teachers' goals matter for professional vision in the first place, then we would expect to find the effects of student‐oriented goals in particular.

Our first research question addressed which goals teachers pursue when watching a video stimulus of classroom instruction. While past work on goals directed at students and the classroom typically focused on mastery and performance goals (Daniels et al., 2013; Daumiller et al., 2022), we note that performance goals may be hard to pursue when watching a video stimulus (in which students' competence is not put in relation to others), while further goals that do fall into a classic achievement goal conceptualization (e.g. Butler, 2007) might also be relevant. Therefore, we take a broad perspective, expecting achievement goals such as mastery goals, while also including further types of goals on an exploratory level.

Our second research question was how such goals are associated with noticing processes in the form of the number and duration of fixations on students. A fundamental assumption was that (more) goals for particular students should go along with more frequent and longer fixations. On an exploratory level, we investigated how different types of goals were related to preservice teachers' attentional processes.

## METHOD

### Sample

The study involved 51 preservice teachers. Of them, 70.6% were female, which is typical for this population in Germany. Their average age was 22.0 years, *SD* = 2.1, and they were, on average, in their 5th semester (SD = 3.1) of their teaching studies. One‐third of them pursued primary education, 67% pursued secondary education: 4% studied the lowest secondary track (Hauptschule), 2% the intermediate secondary track (Realschule), and 61% the highest secondary track (Gymnasium). As such, most preservice teachers had already conducted internships at schools during which they gained practical teaching experience, on average for 36 school days (SD = 27).

### Procedure

Participants were recruited through flyers distributed in main lectures for preservice teachers in two universities in the southern part of Germany, incentivized through a 20 € payment. The students were invited to participate in a video‐based simulation task with their eye movements being recorded. They were guaranteed that their information would be handled according to the data protection regulations of the respective university and analysed for scientific purposes only. They provided informed consent prior to participation. The study was conducted fully in line with the Ethical Principles of Psychologists and the Code of Conduct of the American Psychological Association (American Psychological Association, 2017).

During the video‐based simulation task, participants observed a video stimulus that featured a scripted mathematics lesson for an 11th‐grade class at a German high‐ability track school (‘Gymnasium’) provided by Lewalter et al., (2020). It depicted a teacher‐centred instructional discussion on trigonometry, involving one teacher and 14 student actors. The video angle captured the frontal view from the teacher's perspective (ego perspective). The video was ~3 min.

Participants were invited to the eye tracking lab for individual data collection. After a baseline questionnaire, they watched the first part of the video to familiarize themselves with the teaching situation. Following this, participants were interviewed and asked to imagine themselves as the classroom teacher, specifying the student‐oriented goals they would have in this situation. The interviews lasted ~3–7 min. Afterwards, the eye tracker was calibrated. Participants then watched the rest of the video while their eye movements were recorded. Immediately after watching the video, participants were asked, ‘How well were you able to immerse yourself in the classroom situation shown?’ and ‘Were you able to gain an insight into the classroom situation and the students?’ Responses on a scale from 1 (*not at all*) to 8 (*to the fullest extent*) showed strong agreement, with mean scores of 6.5 and 6.3, respectively, and a minimum score of 5. Although there are no population norms available for these items, their mean scores still suggest a strong level of reported authenticity regarding the videos, supporting the validity of the paradigm.

### Measures

To assess participants' *goals* after they familiarized themselves with the videotaped classroom situation, we used semi‐structured interviews. Participants were asked to put themselves into the position of the classroom teacher and asked about any goals that they might have for individual students or the whole classroom: ‘Now, we are interested in the goals you would pursue as a teacher in such a situation for the class shown. What student‐related goals would you pursue as a teacher in this situation, specifically regarding the whole class? Do you perhaps also have goals that focus on individual students?’. We used follow‐up prompts (e.g. ‘Can you explain what you mean?’, ‘Say more about that’, ‘Can you explain this again using different words?’) to help participants elaborate and explicate their goals. The interviews were conducted by one of the lead authors and two research assistants. To train interviewers and guarantee comparability, we conducted several mock interviews with all interviewers. Interviewers watched and discussed each other's interview approach to ensure that interviews were conducted in similar ways.

**FIGURE 1:**
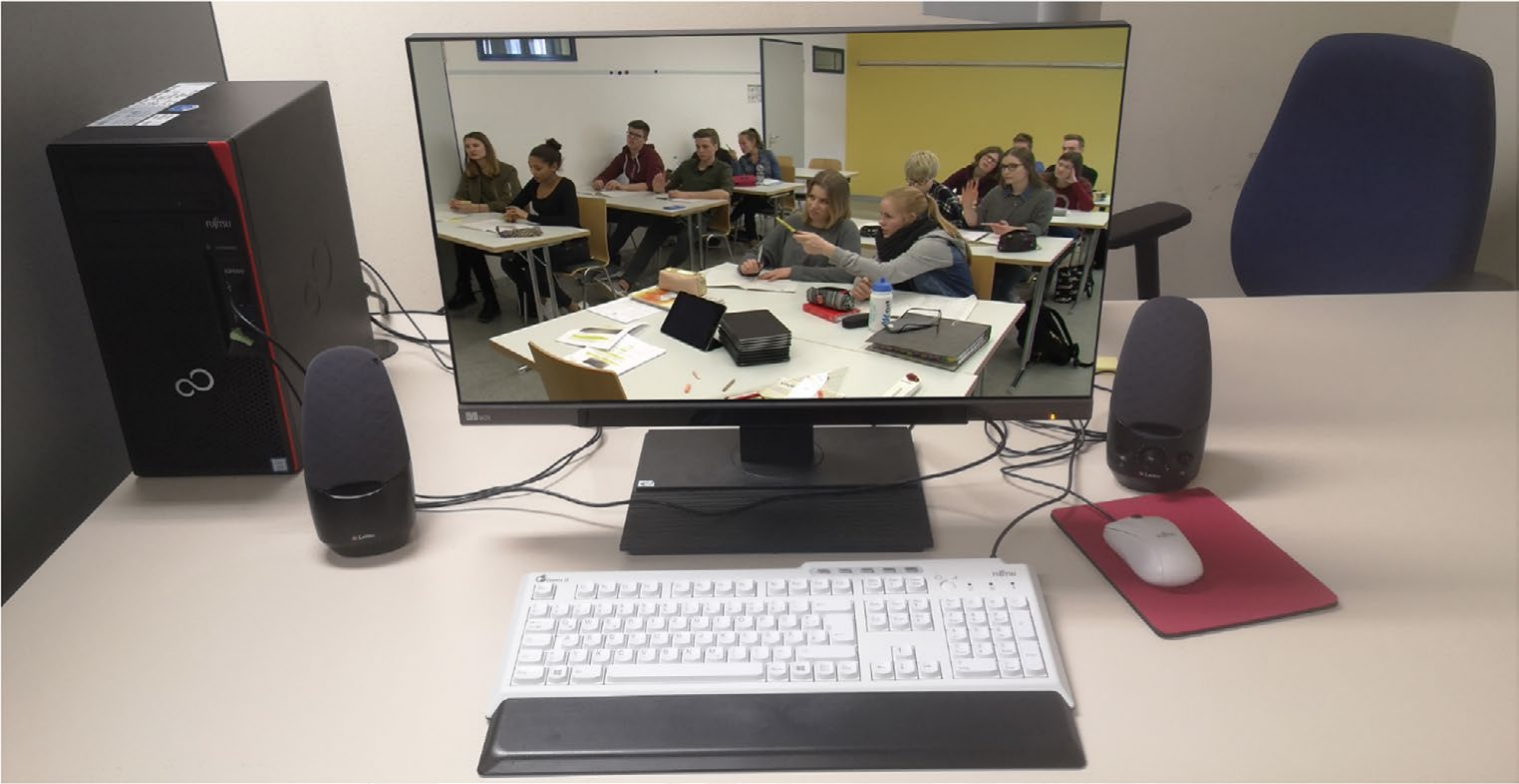
Set‐up of the research design, from the perspective of the research participants. Visible in the background is the chair from which the examiner conducted the interview on participants' goals (they were absent when participants watched the video). The eye tracker is fixed to the bottom of the screen.

To record participants' *noticing* while watching the video scene, a stationary Tobii Pro Fusion Eye Tracker with a 120 Hz sampling rate was used to record their eye movements (see Figure 1 for the research set‐up). The eye tracker was attached to a 24‐inch computer screen. Eye tracking conditions were standardized for all participants and conducted within the universities' eye tracking labs. Before watching the video, each participant was briefed on the eye tracker and positioned within the respective coverage area. Subsequently, a five‐point calibration was performed. To extract eye tracking parameters for individual videotaped students, we defined each student as an area of interest (AOIs). AOIs were transient and of varying size as they were adjusted to students' movements. Eye tracking data for each AOI was aggregated for each participant to determine the sum of the fixation duration and number of fixations for each student in the videotaped classroom. These metrics are commonly used as indicators of cognitive processes that mark attention allocation and depth of information processing (Holmqvist et al., 2011).

### Coding procedure

All interview transcripts were coded using MAXQDA 2024 (VERBI, 2021). Four research assistants categorized participants' articulated goals. First, they coded the goals' targets: goals for the whole classroom or goals directed towards a specific student. Goals directed at subgroups of students (e.g. two students sitting together) occurred to a negligible extent (less than three occurrences in total) and were not pursued further. Each student goal was linked to the respective student identified in the video scene. Next, we used a combination of deduction and induction to develop our coding scheme (see Saldaña, 2016) and identify different types of goals. Initial codes were derived from an instrument developed by Daumiller and Dresel (2020), which classifies common types of achievement goals: mastery goals (including task and learning goals), performance goals (including appearance and normative goals), relational goals, and work avoidance goals. While coding the data, the team met regularly to discuss and refine the instructions and compile a list of further goals. We reached consensus on two additional types of goals that did not fall within the scope of the achievement goal framework: classroom management goals (focused on discipline and behaviour management) and supportive learning climate goals (focused on supportive relationships, social relatedness, respectful interactions, and constructive feedback). Only six goals did not fall into any of these categories, loosely describing cooperation among peers.

The coders were trained in several workshops to discuss the definitions of each goal and to resolve any misunderstandings or disagreements. Initially, the research assistants coded the transcripts independently, and then met for a consensual coding session to discuss and resolve disagreements. The interrater reliability for the independent coding indicated substantial agreement (*κ*_=_ .78). As performance and work avoidance goals were not mentioned at all, the goals considered for the final analysis were mastery goals (primarily encompassing task goals but also learning aspects), relational goals, learning climate goals, and classroom management goals (see Table 1 for an overview).

**TABLE 1:** Overview of the goals articulated in the interviews and their definitions.

Goal type	Focused on	*k*	%	Example excerpts
Goals for individual students				
Mastery	Individual student's task mastery, including competence development and right conduction of tasks	84	62	‘I want to increase her participation in the lesson’, ‘my goal is for him to also experience a sense of achievement’
Relational	Individual student's relationships with peers and teacher	18	6	‘she should feel seen and recognized’, ‘improve her relationship with the teacher’
Learning climate	Individual student's learning atmosphere and sense of belonging	7	22	‘another goal is for the girl in front to have a better solidarity with her neighbours’
Classroom management	Individual student's discipline and organization	10	12	‘I want him to be more attentive’, ‘no more yawning during the lesson’
Total		115	75	
Goals for whole classroom				
Mastery	Whole classroom's task mastery, including competence development and right conduction of tasks	102	96	‘my primary goal is to improve the content knowledge of the whole class’, ‘foster their mathematical competences’, ‘strengthen their self‐concepts’
Relational	Social relationships between classmates and their teacher	10	12	‘my goal is for everyone to feel involved and recognized’, ‘strengthen the social relationships within the whole class’
Learning climate	Inclusive and supportive atmosphere that promotes engagement and motivation among all students	6	18	‘foster a collaborative environment and improve group dynamics, ensuring that students interact and work together effectively’; ‘as another goal, I would set strengthening the class community’
Classroom management	Organized, disciplined, and productive learning environment for the whole classroom	13	24	‘avoid disruptions in the classroom’; ‘ensure that there are no background conversations’
Total		138	100	

*Note*: *k =* total number of articulated goals. % = percentage of participants who reported at least one goal.

### Statistical analysis

To answer our research questions on how teachers' student‐specific and classroom‐related goals guide their visual attention regarding individual students and the whole classroom, we conducted two sets of analyses. First, using two‐level linear modelling, we investigated how teachers' student‐specific goals related to observations of individual students. We modelled the number of student‐specific goals as predictors, and the number and duration of whole fixations per AOI as outcomes. MLR was used as an estimator to account for non‐normal data distribution.

Second, on the aggregated level of participants, we estimated a multivariate regression investigating how teachers' classroom goals are related to their observations of the whole classroom. Therein, we modelled the number of classroom‐specific goals as predictors, and the average number and duration of whole fixations of all students as outcomes.

We conducted these analyses once with the total number of goals as well as, on an exploratory level, differentiating between different types of goal content (distinguishing mastery, relational, learning climate, and classroom management goals). As additional robustness checks, we (1) ran the analyses with dichotomized goals (reflecting whether participants had any goal or none), and (2) included how salient a particular student's behaviour was (classified based on ratings of the video regarding how often a student spoke to the teacher, chatted with classmates, and raised their hand). WLSMV was used as an estimator to account for the ordinal nature of the number of goals. All analyses were conducted with lavaan 0.6‐18 in R 4.4.1 (R Core Development Team, 2023; Rosseel, 2012).

## RESULTS

Participants articulated several student‐oriented goals, directed at both individual students and the whole classroom: a total of 115 goals for individual students and 138 goals for the whole class (see Table 1). All mentioned at least one classroom goal, with a maximum of six goals; student‐specific goals were stated by three out of four. Among them, the most frequent types of goals were mastery goals (*k* = 84 equivalent to 61% of all articulated goals); classroom management goals, learning climate goals, and relational goals were mentioned less frequently (each less than 16% of these goals). This distribution was similar regarding goals for the whole class, with each participant reporting at least one classroom goal, the majority of which were mastery goals (*k* = 102, 74% of all classroom goals mentioned), with classroom management goals, learning climate goals, and relational goals also being mentioned, each to a roughly similar extent.

On average, a participant reported two goals directed at individual students and three goals directed at the whole classroom, with substantial differences between the participants (reflected in substantial standard deviations, see Table 2). A noteworthy finding is that the number of articulated goals was not statistically significantly associated with participants' teaching experience. Next, we investigated whether these interindividual differences in pursued goals were associated with differences in participants' attentional processes in terms of the number and duration of fixations on the students in the classroom.

**TABLE 2:** Descriptive statistics and bivariate correlations.

	Total number of goals		Bivariate correlations^a^			
	*M*	*SD*	Number of fixations per student	Duration of fixations per student	Total number of fixations on students	Average duration of fixations on students
Goals for individual students						
Total	2.1	2.1	.**16**	.**22**	−.05	.08
Mastery	1.5	2.0	.**17**	.**21**	.07	.17
Relational	.1	.6	−.02	−.02	**−.35**	−.22
Learning climate	.2	.5	−.03	−.02	−.15	.05
Classroom management	.3	.7	.08	.**11**	.05	−.10
Goals for whole classroom						
Total	2.7	1.2	−.22	−.16	−.16	−.22
Mastery	2.0	.9	−.21	−.16	−.16	−.21
Relational	.1	.3	−.01	−.21	−.21	−.01
Learning climate	.3	.5	.06	.**29**	.**29**	.06
Classroom management	.2	.5	−.17	−.16	−.16	−.17

*Note*: *N =* 816 student observations within *N* = 51 preservice teachers. Gender is coded 0 = male, 1 = female.

^a^ Presented are Spearman correlations. Correlations between goals for individual students and number and duration of fixations per students are calculated on the level of the individual student observations, all other correlations are calculated on the aggregated participant level. Statistically significant (*p* < .05) correlations are boldfaced.

**FIGURE 2:**
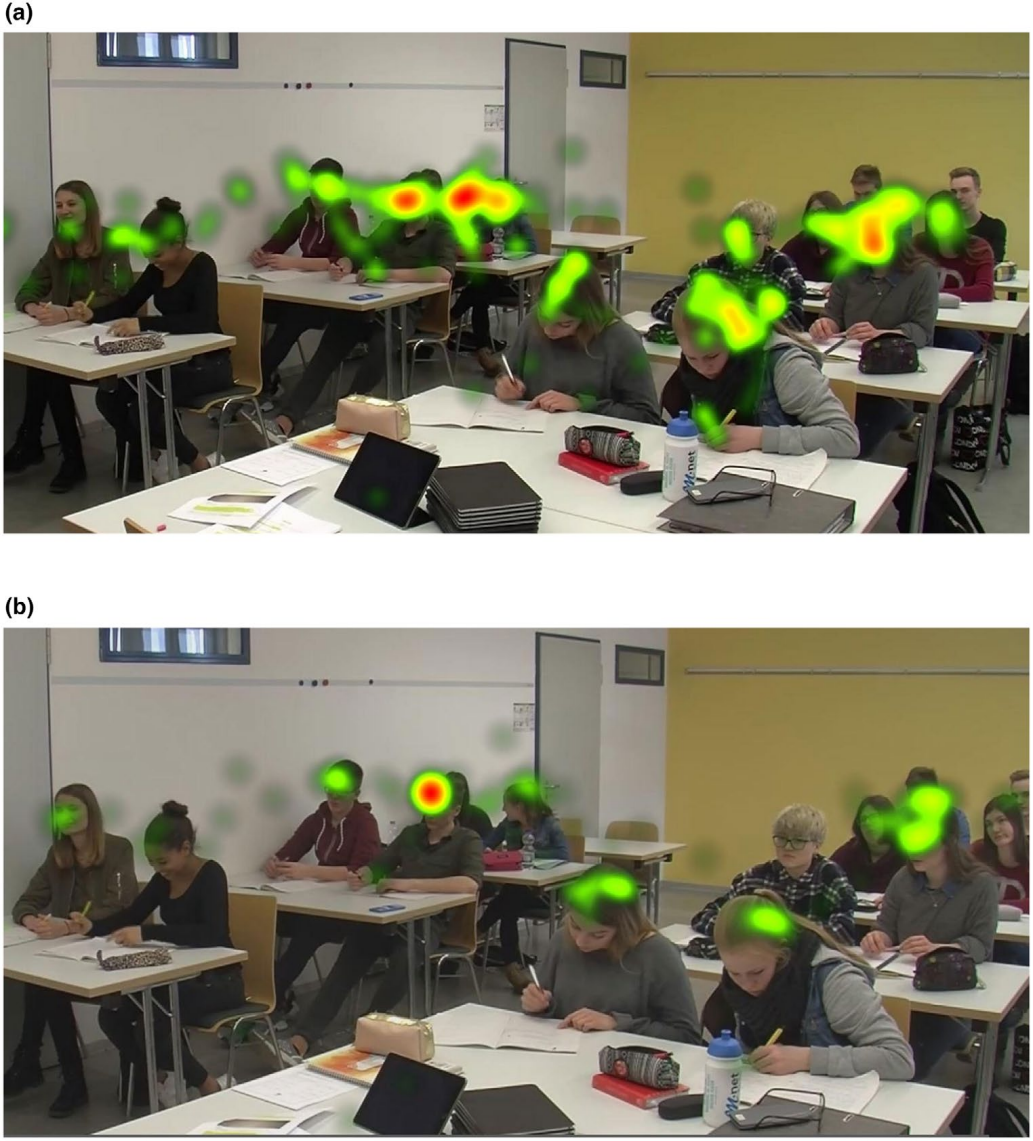
Exemplary heat maps of a participant with two classroom goals (Panel a) and a participant with one classroom goal and three student‐specific goals, of which two were directed at the fourth student from the left (Panel b). Warmer colours represent longer duration of fixations.

Regarding student‐specific goals (see Table 3), we found that the more goals participants reported for a particular student, the more often and the longer they looked at this student (see Figure 2 for an example). Interpreting this, it should be considered that on the level of the individual students, number, and duration of fixations were strongly correlated (*r* = .80). Our explorative analyses based on the different types of pursued goals showed that the observed linkages were particularly driven by student‐oriented mastery goals—associations with relational goals and learning climate goals were statistically non‐significant and descriptively very small.

**TABLE 3:** Associations between student‐specific goals and attention on individual students.

Goals for individual students	Number of fixations per student		Duration of fixations per student	
	*β*	*SE*	*β*	*SE*
Model 1 (Main model)				
Total	.**19**	.04	.**24**	.04
Model 2 (Explorative model)				
Mastery	.**18**	.04	.**22**	.04
Relational	.03	.04	.01	.05
Learning climate	−.01	.03	−.01	.03
Classroom management	.07	.04	.**13**	.06

*Note*: *N =* 816 student observations within *N* = 51 preservice teachers. Presented are standardized regression coefficients. Goals are included and reported as predictors on the within level. ICCs are .05 for duration of fixations, .01 for number of fixations, and .09 for total number of goals. Statistically significant (*p* < .05) values are boldfaced. Statistical significance of the explorative model is tested on a two‐sided level.

For classroom goals (see Table 4), we found that the more goals the participants pursued, the less time they spent fixating on individual students. Descriptively, the number of goals also went along with fewer average fixations; however, this trend did not reach statistical significance (*p* = .05). In contrast to the level of the individual videotaped students, the number and duration of fixations were only weakly associated (*r* = .37). Similar to the student‐specific goals, our explorative analyses indicated that this finding was likely primarily driven by mastery goals, however, their statistical effect was only marginally significant (*p* = .07). Further, and in contrast to this overall pattern, learning climate goals went along with statistically significantly more fixations—providing another indication for different effects depending on the types of goals.

**TABLE 4:** Associations between classroom goals and classroom attention.

Goals for whole classroom	Average number of fixations on students		Average duration of fixations on students	
	*β*	*SE*	*β*	*SE*
Model 1 (Main model)				
Total	−.16	.13	**−.22**	.13
Model 2 (Explorative model)				
Mastery	−.14	.14	−.22	.12
Relational	−.15	.16	.03	.18
Learning climate	.**30**	.11	.07	.12
Classroom management	−.20	.17	−.21	.17

*Note*: *N =* 51 preservice teachers. Presented are standardized regression coefficients. Statistically significant (*p* < .05) values are boldfaced. Statistical significance of the explorative model is tested on a two‐sided level.

Finally, our additional analyses (Tables S1 and S2 in the supplementary materials) indicated that these findings were robust (with descriptively even slightly larger effect sizes) when investigating whether participants had any goal for a particular student or not (instead of the number of goals). Also, when controlling for how salient an individual student behaviour was (i.e. whether they raised their hand, asked a question, or chatted with their classmates), the observed effects were robust. The consideration of the saliency of students' behaviour also proved informative on the classroom level: the more classroom goals were pursued, the shorter and fewer were the fixations on students who behaved more saliently than their peers. Conversely, the relationship between learning climate goals and the average number of fixations was stronger for students who behaved less saliently, meaning that especially for these students, more fixations were registered.

## DISCUSSION

Against the background of the general relevance of teacher motivation for teaching behaviours and the scarce knowledge of the involved processes, we sought to explore their cognitive links, investigating how teachers' student‐oriented goals influence visual attention in classroom settings. In doing so, we addressed a critical gap in the intersection of teacher motivation and teacher professional vision. Through eye tracking methodology, this study provides first, process‐oriented data on the attentional patterns of preservice teachers as they observed a simulated classroom interaction. Our findings confirm the utility of such a paradigm and reveal that goals directed at individual students significantly increase visual attention towards those students, while classroom‐wide goals result in a more dispersed distribution of visual attention. This offers valuable insights into the cognitive mechanisms underpinning effective teaching practices.

### Which goals are pursued when watching the video stimulus?

In our first research question, we were interested in which goals are pursued by participants when watching the classroom instruction. Note that the present study provides a first and particularly critical test of the involved paradigm. Conducted with preservice teachers observing a video in a lab, the study's context is restricted in that participants cannot interact with students or fully grasp their progress, nor were they familiar with the learning biographies of the students shown. Despite this, our findings confirm that preservice teachers already do articulate student‐oriented goals in such a scenario, with large interindividual differences that underscore their relevance.

Mastery goals were frequently articulated, reflecting the typically strong pursuit of such goals among teachers (e.g. Butler, 2007; Daumiller et al., 2022). Despite this commonality, significant interindividual differences were also observed, emphasizing the diverse motivational foci among participants. Interestingly, performance goals were notably absent. This absence may be attributed to the setting of the video stimulus, which does not provide opportunities for students' performance to be directly observable or comparable. Performance goals may necessitate actual classroom interactions where individual or class performance can be contrasted.

Further, work avoidance goals were logically absent as it is not conceivable that teachers set the goal for their students to avoid effort. Moreover, we observed the articulation of additional goals such as classroom management goals and learning climate goals. These findings suggest that in investigating the pathway from teachers' motivation, particularly their self‐directed achievement goals, to their teaching behaviours, it may be helpful to consider a broader range of professional goals than those falling within common achievement goal frameworks (e.g. Butler, 2007; Daumiller et al., 2019; Nitsche et al., 2011). Teachers' motivation likely influences not only the achievement goals they set for their students but also other professional goals affecting their professional vision (Blömeke et al., 2015; Schoenfeld, 2011). The achievement goal approach to motivation emerged from two contrasting views on competence that characterize students' experiences in the classroom achievement context. While mastery and performance goals also matter for teachers—and how they perceive and orient themselves in their achievement context of teaching—for the immediate process of guiding their attention, our findings imply that other goals might matter likewise. In fact, it may not be so much these two different standards on competence inherent in goal pursuit, but possibly (rather, or also) other features, such as their salience or their specificity towards individual students versus the classroom that are shaping different noticing patterns.

Overall, our findings highlight the complexity and diversity of goals that preservice teachers articulate, even in controlled, non‐interactive settings. Future research should continue to explore these dynamics in more naturalistic settings, potentially incorporating mobile eye tracking in real classrooms or virtual reality simulations to better capture an authentic teaching environment.

### How do goals directed at students and the classroom affect their professional vision?

Our second research question was how such goals affect teacher noticing in terms of their number and duration of fixations on students. Our fundamental assumption that goals for individual students should correspond with increased attention on these students was confirmed: effects were observed for both number and duration of fixations, as well as based on whether they had any goal and the number of goals. This suggests that goals have a meaningful impact on the intentional, top‐down processes that guide preservice teachers' attention. In line with research on teacher professional vision, this suggests that goals, as an aspect of teacher motivation, represent a relevant facet contributing to teachers' visual expertise that helps explain attentional processes in specific instructional situations. On a theoretical level, these findings inspire revisions of cognitive models of professional vision to include teacher goals as a driver for the noticing process (Gegenfurtner et al., 2023; Seidel et al., 2024). It confirms theoretically assumed yet seldomly investigated links between teacher motivation and teacher noticing captured via eye tracking methodology (e.g., Blömeke, 2024; Schoenfeld, 2011; Seidel et al., 2024; van Es & Sherin, 2021). To this end, it should be noted that these results reflect situational teacher goals that were articulated for students in a specific classroom situation. Along these lines, and based on a case study with mobile eye tracking, Haataja et al. (2019) found that teachers' situational intentions (in their study, pedagogical intentions) similarly had a significant effect on teachers' selective attention during classroom interaction.

Interestingly, these effects were primarily attributable to mastery goals (see also Chaudhuri et al., 2022). While we are hesitant about statements regarding the effects of goals that were only rarely pursued (due to the limited power involved), this still provides an indication that different types of goals elicit different allocations of attentional resources that should be followed up on in future research. Theoretically, it makes sense that especially mastery goals lead to more and longer fixations: investigating the pursuit of such goals—focused on students' task mastery, learning progress, and development—likely requires more frequent and longer observations than for goals focused on more overt behaviours like rule adherence (Chaudhuri et al., 2022; Haataja et al., 2019; Keskin et al., 2024). Note that the interview approach in the present research was very adequate to investigate the existence and relevance of these goals; however differential effects of different types of goals could only be explored. Future research employing closed response scales and/or an experimental manipulation thereof is needed to better understand how different types of goals affect professional vision.

As classroom goals are directed at all students, we assumed that these would relate to an extended visual field wherein students are fixated on more often but with a shorter duration. Indeed, we found that if preservice teachers pursued more classroom goals in the situation, they spent less time on individual students. Having several competing goals seems to limit the amount of time that is allocated to individual students as preservice teachers focus their attention on getting an overview of the entire classroom that may result in more parafoveal processing (not captured by the eye tracker). Interestingly, this negative effect was primarily evident for students who showed salient behaviours (and for which we also found fewer fixations), but not for those who were passively involved. Considering that novice teachers' attentional processes tend to be driven bottom‐up by salient student behaviour (Gabel et al., 2024; Goldberg et al., 2021), our findings seem to suggest that these salient behaviours that usually attract attention may have been conflicted or crowded out by top‐down mechanisms related to preservice teachers' intentional goal setting (Schütz et al., 2011). In other words, the less classroom goals the preservice teachers pursued, the more strongly were they driven by bottom‐up processes, focusing on the saliently behaving students. Although the conscious selection of relevant visual information is usually a skill attributed to expert teachers (Gegenfurtner et al., 2023; Seidel et al., 2024), these results suggest that goal setting in preservice teachers can affect selective information processing by deciding between goal‐relevant and goal‐irrelevant information. Thereto, they might be highly relevant for further training purposes in teacher education.

### Limitations

Interpreting the results at hand, and considering future research directions, there are a couple of aspects that need to be borne in mind.

First, while the use of interviews to capture participants' goals is a significant strength—offering more concrete insights compared with attributive measures that often fail to reflect the actual presence of such goals—it also introduces a potential bias towards socially desirable responses. This might explain the predominance of mastery goals in our findings, with hardly any performance or avoidance goals reported. Additionally, the power to detect effects for less frequently mentioned goals was limited, making it harder to draw definitive conclusions about their impact.

Second, the study's ecological validity may be questioned, as observing a video stimulus differs from experiencing a live classroom environment. Despite participants rating the authenticity of the videos and their immersion as highly immersive, the dynamic nature of an actual classroom could influence teacher' goals and visual attention differently. Even though the video stimulus showed a typical teacher‐centred classroom situation, it is important to note that goals were articulated in response to the specific student behaviour depicted in the video. Analysing additional classroom scenes could help clarify the extent to which the number and type of goals are influenced by the nature and characteristics of the classroom context.

Third, our sample consisted of preservice teachers whose levels of professional knowledge differ from those of more experienced in‐service teachers. Subsequent studies should consider including a diverse range of participants with varying levels of teaching experience to enhance the generalizability of the results. Especially comparisons between experts and novices, as commonly done in research on teacher professional vision (Grub et al., 2020; Keskin et al., 2024; Stahnke et al., 2016), might be a rewarding direction for future research to better understand how the observed processes might depend on differences in experience and professional knowledge. Particularly interesting seems in‐service teachers' daily experience with their students: as they typically know their students' learning biographies and typical classroom behaviour very well, associations between student‐specific goals might relate to fixations in the classroom differently. Relatedly, future studies could explore the role of teachers' school subjects to better understand potential subject‐specific patterns in teachers' goal setting. Goals directed at individual students or the whole classroom may differ for teachers from various subject domains**—**especially when their subject may not be aligned with the subject depicted in the classroom video. Although we do not assume subject‐specific patterns for the relation between preservice teachers' situation‐specific goals and their attention towards individual students, future studies need to investigate the consistency of these effects across multiple samples of teachers.

Fourth, another limitation is that while we gained valuable data on the visual focus of participants, we did not capture the specific cognitions accompanying their observations of the students. Future research should address this gap, for example by examining participants' reasoning about their attention allocation. This might be facilitated through think‐aloud protocols or retrospective interviews based on gaze replays (Stahnke & Gegenfurtner, 2024). Such an approach would offer a richer understanding of how goals influence not only visual attention but also the cognitive processing of classroom interactions, providing another promising direction for future studies.

Finally, while our innovative and explorative study design makes an important contribution to the field by providing first insights into the importance of teachers' goals for the situational guidance of visual attention in instructional settings, the robustness and generalizability of the link between goal pursuit and teachers' noticing needs to be followed up on with specifically tailored research designs. For example, multi‐trail studies with multiple video stimuli could further examine the consistency and reliability of the current findings across varying contexts. In addition, multi‐group analyses are required to control for teacher characteristics such as teachers' grade level, prior teaching experience or subject domain to determine if patterns vary across different samples.

## CONCLUSION

Our research highlights the influence of teachers' goals in orchestrating the dynamic dance of visual attention within classroom instruction. Whether directed at the entire class or specific students, student‐oriented goals significantly influence the distribution and intensity of visual attention. Goals aimed at individual students lead to more and longer fixations on those students. Conversely, goals targeting the whole classroom seem to result in a more dispersed and bottom‐include – visual attention pattern. Our findings also suggest nuanced effects depending on goal content and the visual saliency of student behaviours, warranting a deeper consideration of the cognitive processes at hand as well as the respective context. Notably, our research illuminates the fruitfulness of a combination of research on teacher motivation and teacher professional vision, along with the utility and applicability of an eye tracking paradigm for delving into the effects of motivation on classroom perception. We believe that this line of research holds promise for advancing our understanding of the cognitive pathways linking teacher motivation to their classroom behaviours—through goal setting and professional vision.

## AUTHOR CONTRIBUTIONS

**M. Daumiller:** Conceptualization; methodology; software; validation; investigation; data curation; formal analysis; writing – original draft; writing – review and editing; project administration; visualization. **R. Böheim:** Writing – original draft; writing – review and editing; project administration; conceptualization; methodology; software; investigation; validation; data curation; visualization. **A. Alijagic:** Validation; methodology; writing – review and editing. **D. Lewalter:** Writing – review and editing; resources. **A. Gegenfurtner:** Writing – review and editing; conceptualization. **T. Seidel:** Conceptualization; methodology; project administration; writing – review and editing. **M. Dresel:** Conceptualization; methodology; writing – review and editing; project administration.

## Supporting information


Data S1

## Data Availability

The data that support the findings of this study are available on request from the corresponding author. The data are not publicly available due to privacy and ethical restrictions.
