# Supplementary material for: Guiding attention in the classroom: An eye‐tracking study on the associations between preservice teachers' goals and noticing of student interactions
Source: Br J Educ Psychol. 2025 Mar 10;95(Suppl 1):S115–32. doi: 10.1111/bjep.12748 (PMC12427153; doi:10.1111/bjep.12748)
Supplement: Supplementary file 1 — Data S1 [file BJEP-95-S115-s001.pdf]

Guiding Attention in the Classroom: An Eye-Tracking Study on the Associations between Preservice Teachers' Goals and Noticing of Student Interactions

M. Daumiller[1,*], R. Böheim[2,*], A. Alijagic[3], D. Lewalter[2], A. Gegenfurtner[3], T. Seidel[2,**], M. Dresel[3,**]

[1] LMU Munich, Germany

[2] Technical University of Munich, Germany

[3] University of Augsburg, Germany

https://doi.org/10.1016/10.1111/bjep.12748

- Supplemental Information -

This document contains materials designed to supplement the main text. The materials include the following:

1. Table S1: Results of Additional Analyses for Associations Between Student-specific Goals and Attention on Individual Students
2. Table S2: Results of Additional Analyses for Associations Between Goals and Classroom Attention

Table S1

Results of Additional Analyses on Associations Between Student-specific Goals and Attention on Individual Students

Goals for individual students	Number of fixations per student		Duration of fixations per student	
	β	*SE*	β	*SE*
Model 3				
Total number of goals	**.16**	**.04**	**.20**	**.05**
Salience of respective students	**.29**	**.03**	**.38**	**.03**
Model 4				
Mastery	**.16**	**.04**	**.19**	**.04**
Relational	.01	.04	–.01	.04
Learning climate	.01	.03	.01	.03
Classroom management	.06	.05	**.12**	**.06**
Salience of respective students	**.29**	**.04**	**.38**	**.03**
Model 5				
Total number student goals (dichotomous)	**.20**	**.05**	**.22**	**.04**

Note. $N = 816$ student observations within $N = 51$ preservice teachers. Presented are standardized regression coefficients. Goals are included and reported as predictors on the within level. Paralleling the main analysis, statistical significance of Model 4 is tested on a two-sided level. Statistically significant ($p < .05$) values are boldfaced. Coefficients that were statistically significant in the main models have gray background.

Table S2

Results of Additional Analyses on Associations Between Goals and Classroom Attention

	Average number of fixations on students				Average duration of fixations on students			
	Non-salient		Salient		Non-salient		Salient	
Goals for whole classroom	β	*SE*	β	*SE*	β	*SE*	β	*SE*
Model 3								
Total number of goals	.01	.13	**–.20**	**.11**	–.07	.13	**–.27**	**.12**
Model 4								
Mastery	.01	.15	–.12	.12	–.17	.14	–.25	.13
Relational	–.02	.13	.16	.18	.17	.14	–.03	.17
Learning climate	**.40**	**.10**	.21	.12	.20	.18	.01	.12
Classroom management	–.22	.13	–.23	.15	–.15	.16	–.19	.16

Note. $N = 51$ preservice teachers and their results for non-salient (averaged from $N = 408$) and salient (averaged from $N = 408$) students. Students' behaviors were classified as salient if they engaged in hand raising, talked to the teacher, or chatted with their peers. Dichotomizing goals on the classroom level did not make sense as almost all participants reported at least one classroom goal. Presented are standardized regression coefficients. Paralleling the main analysis, statistical significance of Model 4 is tested on a two-sided level. Statistically significant ($p < .05$) values are boldfaced. The relationship between mastery goals and average duration of fixations on salient students was marginally significant with $p = .06$. Coefficients that were statistically significant in the main models have gray background.
